# Surgical resection of peripheral odontogenic fibromas in African pygmy hedgehog (*Atelerix albiventris*): a case study

**DOI:** 10.1186/s12917-015-0455-0

**Published:** 2015-07-04

**Authors:** Anna Wozniak-Biel, Maciej Janeczek, Izabela Janus, Marcin Nowak

**Affiliations:** Department of Epizootiology and Clinic of Bird and Exotic Animals, Wrocław University of Environmental and Life Sciences, Wroclaw, Poland; Department of Animal Physiology and Biostructure, Wrocław University of Environmental and Life Sciences, Wroclaw, Poland; Department of Pathology, Wrocław University of Environmental and Life Sciences, Wroclaw, Poland

**Keywords:** African pygmy hedgehog, Peripheral odontogenic fibroma, Oral disorders, Oral tumors and histopathology

## Abstract

**Background:**

Neoplastic lesions of the mammary gland, lymph nodes, or oral cavity in African pygmy hedgehogs (*Atelerix albiventris*) are common in captive animals. Chemotherapy and radiotherapy protocols have not yet been established for the African pygmy hedgehog. Thus, surgical resection is the current treatment of choice in this species.

**Case presentation:**

A 5-year-old male African pygmy hedgehog showed multiple erythematous, round small tumors located in the oral cavity, on both sides of maxilla. The treatment of choice was surgical resection of tumors using a surgical knife under general anesthesia. Excised neoplastic lesions were diagnosed as peripheral odontogenic fibroma by histopathology. Six months after surgery relapse of tumors in the oral cavity was not observed.

**Conclusions:**

The treatment adopted in this case report is safe for the patient and provides the best solution for mild proliferative lesions of the oral cavity. To our knowledge this is the first report of surgical resection of oral tumors (peripheral odontogenic fibroma) in the African pygmy hedgehog.

## Background

The African pygmy hedgehog (*Atelerix albiventris*) has become a very popular pet in Poland in the last few years. It is smaller than the European hedgehog and is a member of the insectivore family *Erinaceidae*, subfamily *Erinaceinae*. African pygmy hedgehogs are domesticated animals and live for about 5–7 years in captivity. They possess 36 brachyodontic teeth: 2(3/2,1/1,3/2,3/3) with the first incisors being notably longer than the rest, and are spaced apart [1].

In studies of hedgehogs at histopathology of surgically resected tumor or necropsy approximately 40 % of hedgehogs aged from 1 month to 3 years were diagnosed with neoplastic disease [2, 3]. The most common histologic types of tumors are mammary gland adenocarcinoma, lymphoma, and oral squamous cell carcinoma [2, 4, 5]. The digestive tract, including the oral cavity, is the third most common site of neoplastic disease in hedgehogs [4]. Chemotherapy and radiotherapy protocols have not yet been established for the African pygmy hedgehog, and therefore, surgical resection is currently the best treatment in cases where the tumor is benign, well separated from the healthy tissue and without metastases.

Medical knowledge, veterinary care, and the awareness of African pygmy hedgehog owners are ever increasing. The average life span of domesticated animals is prolonged compared with wild animals. This situation predisposes domesticated hedgehogs to more frequent development of tumors, including oral cavity. In addition, it is well known that periodontal disease, tooth root abscesses, and various neoplasms (e.g. squamous cell carcinoma, lymphosarcoma) occur frequently in African pygmy hedgehogs >3 years old [4].

Peripheral odontogenic fibroma (previously named as fibromatous epulis of periodontal ligament origin) is a peripheral odontogenic neoplasm, indistinguishable clinically from fibrous hyperplasia, most common in dogs, and rarely occurring in cats. The prognosis following surgical removal is good [6, 7].

To our knowledge, this is the first case report of surgical resection of a peripheral odontogenic fibroma in the African pygmy hedgehog. The significance of this case report is that it will enable veterinary clinicians to familiarize themselves with the surgical resection of benign oral tumors (peripheral odontogenic fibroma) in the African pygmy hedgehog and consider the peripheral odontogenic fibroma as other primary neoplasm of oral cavity in this species.

## Case presentation

**Fig. 1:**
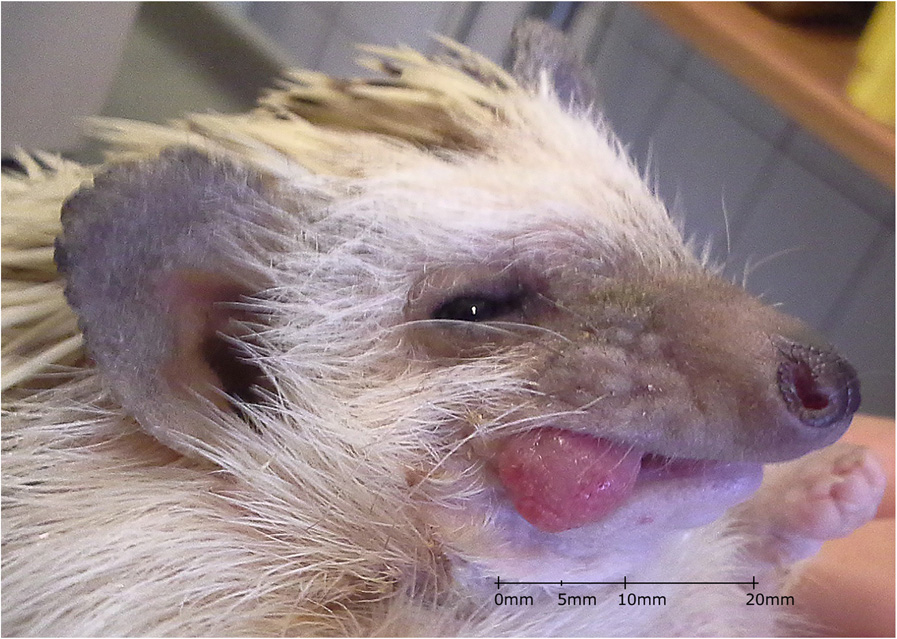
Preoperative view of oral tumors located above the molar teeth, on both sides of the maxilla in an African pygmy hedgehog

**Fig. 2:**
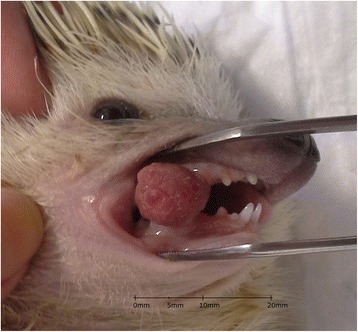
Preoperative view of oral tumors located above the molar teeth, on both sides of the maxilla in an African pygmy hedgehog

**Fig. 3:**
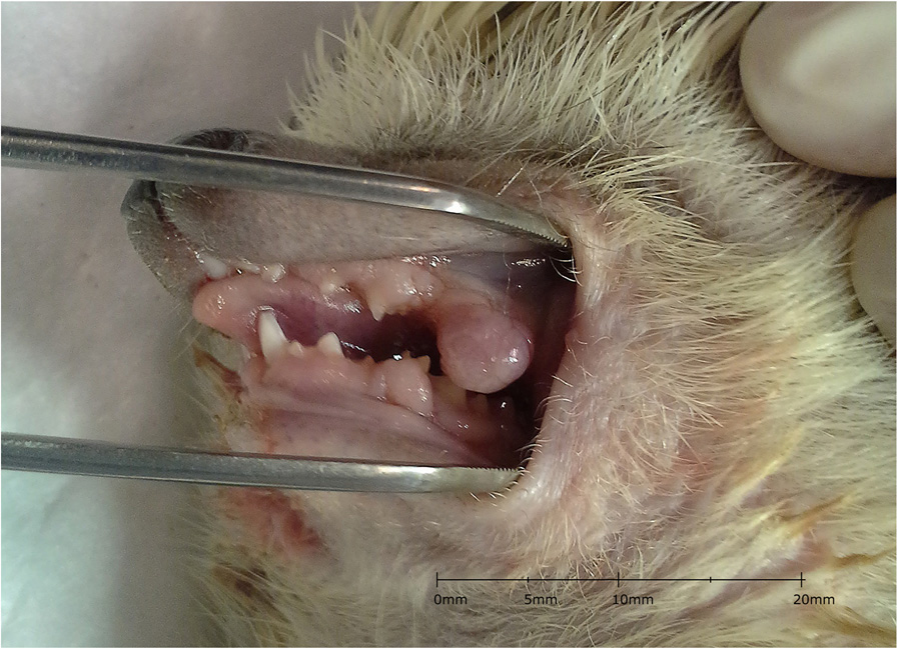
Preoperative view of oral tumors located above the molar teeth, on both sides of the maxilla in an African pygmy hedgehog

A 5-year-old male African pygmy hedgehog showed two erythematous, round small tumors protruding from the oral cavity. One tumor was visible on the closed mouth on the right side of the head (Figs. 1 and 2), and the other was located on the left side, inside the oral cavity, above the molar teeth (Fig. 3). Both tumors were well separated from the gum, pedunculated, soft texture, uneven surfaces, painless. The neoplastic lesion on the left side had a diameter of approximately 5 mm, was pale pink, while the lesion on the right side had a diameter of approximately 11 mm and on its surface small foci of hyperemia were observed. There was no deformation of hard tissue of splanchnocranium during clinical examination. The pet owner complained of problems with food and water intake, because of the neoplastic tumors located on the surface of maxilla. The animal’s appetite remained good. Physical examination revealed the animal was in very good condition with a rectal temperature of 36.8 °C.

**Fig. 4:**
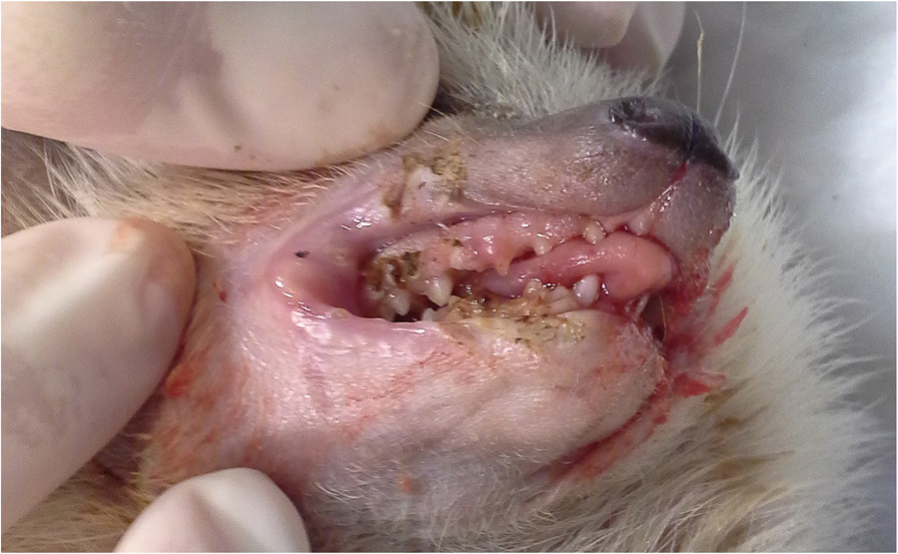
Postoperative view of the excised oral tumors using surgical knife electrocoagulation

**Fig. 5:**
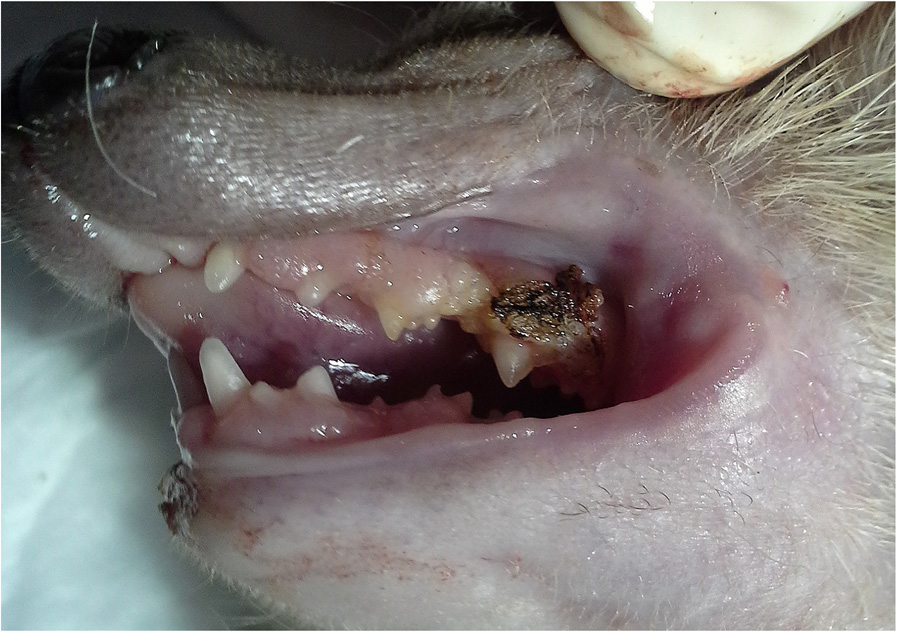
Postoperative view of the excised oral tumors using surgical knife electrocoagulation

Anesthesia was induced with 30 mg/kg of ketamine hydrochloride (Bioketan; Vetoquinol Biowet, Gorzow Wielkopolski, Poland) and 0.15 mg/kg of medetomidine hydrochloride (Domitor; Zoetis, Florham Park, NJ, USA) intramuscularly (IM). To prevent hypersalivation, atropine sulfate (Atropinum Sulfuricum; Polfa, Warszawa, Poland) at 0.03 mg/kg IM was administered. The preferred method for sedating small animals is gas anesthesia with isoflurane or sevoflurane [8], but in this case using a facial mask was impossible because of the location of the tumors. The animal was placed on a heating mat to prevent hypothermia. A portable veterinary monitor (MEC-1200-Vet; Mindray, Shenzhen, China) was used to constantly monitor the patient’s breathing rate and heart rate. Surgical resection of tumors and bleeding were controlled simultaneously using a surgical knife electrocoagulation system (Martin System 2000; Gebrüder Martin GmbH & Co, Tuttlingen, Germany). The results of surgical resection of the oral tumors are shown in Figures (Figs. 4 and 5).

After surgical procedures, atipamezole hydrochloride (Antisedan; Zoetis) 0.3 mg/kg IM and meloxicam (Metacam; Boehringer Ingelheim, Ingelheim, Germany) 0.2 mg/kg subcutaneously were administered [9]. The patient’s condition was monitored until it reached full consciousness.

The excised tumors underwent fixation in 7 % buffered formalin, were embedded in paraffin blocks, and 6 μm slides were cut. The preparations were stained using the standard hematoxylin-eosin method [10], and subsequently evaluated using light microscopy using WHO guidelines for the evaluation of oral cavity tumors. Photomicrographs of the preparations were obtained using computer-amplified image analysis and an optical microscope (Olympus BX53; Olympus, Tokio, Japan). Histopathological analysis was conducted using the cell^^^A software (Olympus Soft Imaging Solution GmbH, Münster, Germany).

## Results and discussion

**Fig. 6:**
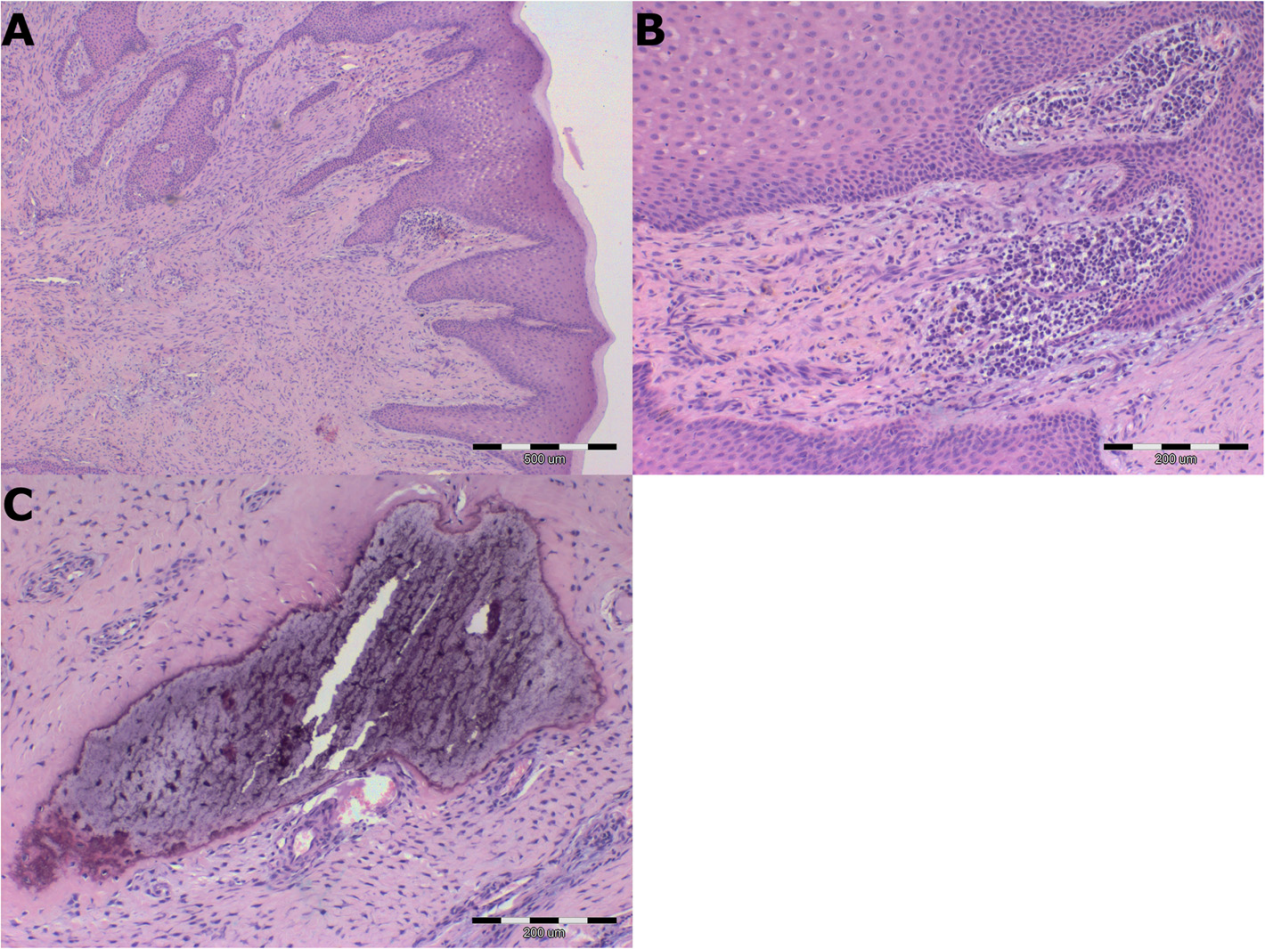
Histopathological pattern of peripheral odontogenic fibroma in the pygmy hedgehog. **a** Small spindle to stellate fibroblasts immersed in eosinophilic dense collagen matrix with branching cords and islands of odontogenic epithelium and peripheral palisading of same epithelium. **b** A small amount of mononuclear inflammatory cells accompanying the peripheral odontogenic fibroma. **c** Areas of hard tissue corresponding with areas of mineralization within the tumor

Histopathological examination of lesions showed epithelial covered, well-vascularized, cellular fibroblastic tissue comprised of small spindle to stellate fibroblasts with small dark basophilic nuclei dispersed in a dense collagen matrix. Few mononuclear inflammatory cells, areas of hard tissue corresponding with areas of mineralization, and branching cords or islands of epithelium with peripheral basal stratum were also observed (Fig. 6a, b, and c). All tumors were removed with an appropriate (approximately 2–5 mm) clinical surgical margin and evaluated later by histopathology. The observed pattern is characteristic for peripheral odontogenic fibroma [6, 7]. Peripheral odontogenic fibromas have been extensively reported in a variety of domestic mammals and humans [11–16]. However, to our knowledge, there is no information concerning peripheral odontogenic fibroma in the African pygmy hedgehog.

According to the literature, the most common tumors of the gastrointestinal tract in hedgehogs are oral squamous cell carcinoma, intestinal adenocarcinoma, acinic cell carcinoma, metastatic hepatocellular carcinoma, fibrosarcoma, plasmocytoma, and lymphoma [4, 17–19]. Raymond and Garner [4] diagnosed 35 (53 %) neoplasms in a group of 66 hedgehogs. In three of the 35 animals, more than one type of tumor was present. The authors revealed that 85 % of the tumors were malignant [4]. Raymond et al. [20] indicated the presence of malignant mast cell tumor in adult African hedgehog. This tumor was located subcutaneously, along to ventral part of the neck with metastasis to local lymph node [20]. In other studies, the authors revealed the evidence of a probable retrovirus associated with multicentric sarcomas in two 3 years old hedgehogs, male and female [21]. In our case, the investigated tumor was benign. Nonetheless, its location in the oral vestibule, which caused the animal’s discomfort, pain, and risk of hemorrhage, was an indication for surgery.

## Conclusions

An early and accurate diagnosis is essential for positive prognosis, curative treatment, and fast recovery in hedgehogs. The resection of oral cavity tumors in the African pygmy hedgehog carried out in this case report can be successfully applied by veterinary clinicians. The established protocol is safe for the patient and provides the best solution for mild proliferative lesions of the oral cavity. To our knowledge this is the first report of surgical resection and histological description of oral tumors (peripheral odontogenic fibroma) in the African pygmy hedgehog.
